# Episodic ataxia and severe infantile phenotype in spinocerebellar ataxia type 14: expansion of the phenotype and novel mutations

**DOI:** 10.1007/s00415-021-10712-5

**Published:** 2021-07-22

**Authors:** Giovanna De Michele, Daniele Galatolo, Serena Galosi, Andrea Mignarri, Gabriella Silvestri, Carlo Casali, Vincenzo Leuzzi, Ivana Ricca, Melissa Barghigiani, Alessandra Tessa, Ettore Cioffi, Caterina Caputi, Vittorio Riso, Maria Teresa Dotti, Francesco Saccà, Giuseppe De Michele, Sirio Cocozza, Alessandro Filla, Filippo M. Santorelli

**Affiliations:** 1 Department of Neurosciences, Reproductive and Odontostomatological Sciences, Federico II University, Via Sergio Pansini 5, 80131 Naples, Italy; 2 Istituto Di Ricovero E Cura a Carattere Scientifico (IRCCS), Fondazione Stella Maris, Pisa, Italy; 3 Department of Human Neuroscience, Sapienza University of Rome, Rome, Italy; 4 Department of Medicine, Surgery and Neuroscience, Neurology and Neurometabolic Unit, University of Siena, Siena, Italy; 5 Department of Neurosciences, Faculty of Medicine and Surgery, Catholic University of Sacred Heart, Rome, Italy; 6 Neurology Unit, Fondazione Policlinico Universitario A. Gemelli, IRCCS, Rome, Italy; 7 Department of Medical and Surgical Sciences and Biotechnologies, Sapienza University of Rome, Rome, Italy; 8 Department of Advanced Biomedical Sciences, Federico II University, Naples, Italy

**Keywords:** Spinocerebellar ataxia type 14, *PRKCG*, NGS targeted resequencing panel, Novel mutations, Broadened phenotype

## Abstract

**Introduction:**

Spinocerebellar ataxia type 14 (SCA14) is a dominantly inherited neurological disorder characterized by slowly progressive cerebellar ataxia. SCA14 is caused by mutations in *PRKCG,* a gene encoding protein kinase C gamma (PKCγ), a master regulator of Purkinje cells development.

**Methods:**

We performed next-generation sequencing targeted resequencing panel encompassing 273 ataxia genes in 358 patients with genetically undiagnosed ataxia.

**Results:**

We identified fourteen patients in ten families harboring nine pathogenic heterozygous variants in *PRKCG*, seven of which were novel. We encountered four patients with not previously described phenotypes: one with episodic ataxia, one with a spastic paraparesis dominating her clinical manifestations, and two children with an unusually severe phenotype.

**Conclusions:**

Our study broadens the genetic and clinical spectrum of SCA14.

**Supplementary Information:**

The online version contains supplementary material available at 10.1007/s00415-021-10712-5.

## Introduction

**Fig. 1:**
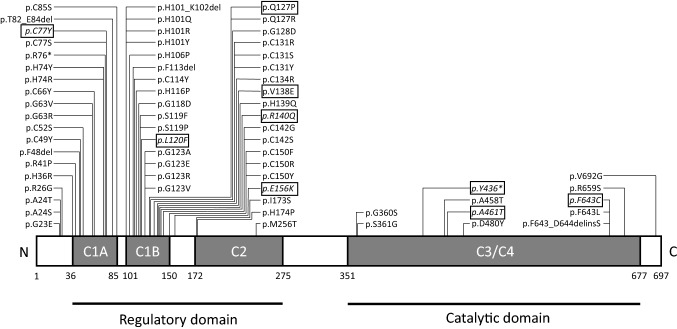
Variants associated with ataxia so far described in the protein kinase C γ coded by *PRKCG*. Pathogenic or likely pathogenic variants identified in this study are boxed (novel ones are indicated in italics). It should be noticed that the splicing variant c.285C > G and two large-scale deletions previously associated with SCA14 are not reported in this figure. Legend: 1A and C1B = cysteine-rich regions, C2 = Ca^2+^ sensitive region, C3 = kinase region, C4 = substrate recognition region

Spinocerebellar ataxia type 14 (SCA14, OMIM 605,361) is a rare disorder with a frequency varying from 1 to 7% of all non-polyQ autosomal dominant cerebellar ataxias [1–4]. It is caused by mutations in the *PRKCG* gene coding for the protein kinase C gamma (PKCγ), an isoform of the protein kinase C family exclusively expressed in neurons and highly expressed in Purkinje cells. PKCγ comprises a regulatory domain, where lay most mutations so far described, and a catalytic domain, where pathogenic variants are reported to cause a more complicated phenotype [5, 6]. To date, almost all reported disease-causing variants are missense, a single nonsense mutation has been described [7] (Fig. 1).

SCA14 phenotype is that of a slowly progressive ataxia, rarely associated with severe disabilities. The average onset occurs in the thirties, but is highly variable, from childhood to seventh decade. Dysarthria, dysmetria, abnormal ocular movements (nystagmus mainly) are present in most patients. Increased knee jerks, rarely associated with Babinski signs, are present in one third. Myoclonus and dystonia are described in a minority. Cognitive impairment is rarely present and is usually mild, but may range from a dysexecutive syndrome to overt dementia. Other features include depression, tremor or titubation, rarely hypoacusia, parkinsonism, psychosis, and epilepsy [2, 4, 5]. Peripheral nerve conduction studies are usually normal [3]. MRI shows a variable atrophy of the cerebellum, more marked in the vermis, without other abnormalities in brainstem and cerebral cortex [3].

We present fourteen patients in ten families harboring nine pathogenic heterozygous variants in *PRKCG*, seven of which were novel. We encountered four patients with not previously described phenotypes: one with episodic ataxia, one with a spastic paraparesis dominating her clinical manifestations, and two children with an unusually severe phenotype.

## Patients and methods

In a multicenter Italian study addressing the etiology of inherited ataxias and related disorders, we investigated 358 index patients with genetically undiagnosed ataxias, both familial (17%) and sporadic (83%) from 16 neurological centers belonging to ITASPAX network using a targeted resequencing multigene panel (TRP) in next-generation sequencing (NGS). This study was approved by Local Ethics Committees. All the participants (or their legal guardians in case of children) provided written informed consent, according to Italian National Health System guidelines and Declaration of Helsinki, to participate to the study as described before [8]. Briefly, a TRP (SureSelect, Agilent, Santa Clara, CA) encompassing 273 genes related to inherited ataxias was employed, and sequencing was carried out using a NextSeq 500 (Illumina, San Diego, CA) platform (see Supplementary Table 1). Variant annotation was made using the Ingenuity Variant Analysis suite (Qiagen, Hilden, Germany) according to an in-house validated pipeline [9]. All variants identified were confirmed by Sanger Sequencing. Prior to this study, all patients had been tested for pathological trinucleotide repeat expansions associated with FRDA and SCA1, 2, 3, 6, 7, 17 and *FMR1*. Exome sequencing in families I and J (trio analysis) was performed as described previously [8].

The patients were evaluated by SARA (Scale for the Assessment and Rating of Ataxia), that ranges from 0 (no ataxia) to 40 (severe ataxia) [10], and by disability scale SDFS (Spinocerebellar Degeneration Functional Score), that spans from 0 (no disability) to 7 (bedridden) [11].

The progression rate was calculated dividing SARA score at last examination by years of disease duration.

Examined patients underwent a conventional brain MRI scan with acquisition of T1-weighted, T2-weighted and Diffusion Weighted (DWI) images along the three orthogonal planes. All scans were acquired at the Neuroradiology Department of respective referral centers using a high field scanner (≥ 1,5 Tesla). Images were qualitatively evaluated, regarding the presence and pattern of cerebellar and cerebral atrophy (reported as absent, mild, moderate and severe), as well as the presence of both infra- and supratentorial T2-weighted signal changes.

Neurophysiological studies were performed in selected patients.

## Results

**Fig. 2:**
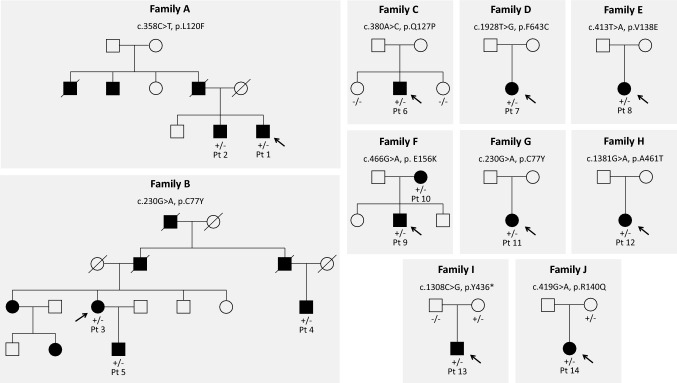
Family trees of the ten kindreds. Boxes are men and circles women. Full symbols indicated affected individuals. Empty symbols indicated not tested or wild-type relatives

Overall, our TRP study yielded a 34% diagnostic yield (manuscript in preparation) a finding in line with similar size studies. In 2.8% of the whole cohort (10 index cases among 14 patients overall, one of whom (Pt. 6) already reported [12]), we identified nine different variants in the *PRKCG* gene, seven of which were novel. They occurred in 10 kindred with two unrelated families (families B and G) carrying the same mutation (c.230G > A, p.C77Y). Segregation studies were available in five kindred (families A, B, F, I and J). The transmitting parent was the father in family A and the mother in the remaining families (Fig. 2). American College of Medical Genetics and Genomics published guidelines [13] were used for pathogenicity scoring. To determine the effect of missense mutations on protein integrity, we employed an in silico pipeline embracing fourteen algorithms (CADD, https://cadd.gs.washington.edu/snv; SIFT, https://sift.bii.a‐star.edu.sg/; PolyPhen-2, http://genetics.bwh.harvard.edu/pph2/; MutationTaster, http://www.mutationtaster.org/; FATHMM-MKL, http://fathmm.biocompute.org.uk/fathmmMKL.htm; REVEL, https://sites.google.com/site/revelgenomics/; MutPred, http://mutpred.mutdb.org/; Mutation Assessor, http://mutationassessor.org/r3/; Provean, http://provean.jcvi.org/index.php; UMD-Predictor, http://umd-predictor.eu/; MetaSVM, MetaLR, GERP +  + , PrimateAI, https://varsome.com/). Almost all converged to assess the pathogenic effect of variants identified (see Supplementary Table 2). Moreover, novel mutations were either absent or ultra-rare in gnomAD (https://gnomad.broadinstitute.org/) and affected residues highly conserved through evolution in multiple species. Six variants laid in the PKCγ regulatory domain, three in the catalytic part of the protein. Novel variants recur in known mutational protein domains, and two (p.C77Y and p.F643C) hit the same residue of previously described disease-associated variants. Finally, c.1308C > G, p.Y436*, found in family I, was the second nonsense mutation so far described in *PRKCG* (Fig. 1).

The main clinical features of the fourteen patients are summarized in Table 1. The mean age of onset ± SD was 32.6 years ± 19.3, with a range from 0 to 66. Two patients had onset in the first year of life. Mean age at diagnosis was 46.3 years ± 18.7. Age at last examination was 46.6 years ± 18.6, with a mean disease duration of 14.0 years ± 11.1. The mean SARA score was 13.6 ± 9.7 (range 2–31). Only one patient was wheelchair-bound, after 8 years from onset. Mean progression rate (SARA score per year) was 1.23 ± 0.96. Mean SDFS score was 2.7 ± 1.2.

**Table 1:** Clinical and MRI findings

Patient, Family	Sex	Onset (yrs)	Age (yrs)	Duration (yrs)	SARA score	SDSF	Gait ataxia	Dysarthria	Dysmetria	Abnormal ocular movements	Knee jerks	LL increased tone	Babinski signs	LL decreased vibration sense	Dystonia	Tremor	Cognitive impairment	MRI Cerebellar atrophy	*PRKCG* mutation
1, A	M	44	51	7	8	2	Yes	Yes	Yes	SP	Weak	No	No	Yes	No	No	No	+ + +	c.358C > T, p.L120F
2, A	M	50	56	6	6	2	Yes	Yes	Yes	SP	Weak	No	No	Yes	No	No	No	+ +	c.358C > T, p.L120F
3, B	F	40	71	31	22	3	Yes	Yes	Yes	No	Normal	No	No	No	No	No	No	+	c.230G > A, p.C77Y
4, B	M	56	69	13	22	3	Yes	Yes	No	No	Normal	No	No	No	No	No	Mild	+	c.230G > A, p.C77Y
5, B	M	21	41	20	19	3	Yes	Yes	Yes	No	Normal	No	No	No	No	No	ID	+	c.230G > A, p.C77Y
6, C	M	30	41	11	26	6	Yes	Yes	Yes	SS	Normal	No	No	No	Yes	Yes	Mild	+	c.380A > C, p.Q127P
7, D	F	16	58	42	14	3	Yes	Yes	Yes	Ny	Normal	No	No	No	No	No	No	+ +	c.1928 T > G, p.F643C
8, E	F	35	42	7	5	2	Yes	Yes	No	No	Normal	No	No	No	No	No	No	+ +	c.413 T > A, p.V138E
9, F	M	24	40	16	8	2	Yes	Yes	Yes	HS	Brisk	Yes	Yes	No	No	No	No	+ +	c.466G > A, p. E156K
10, F	F	66	67	1	2	1	Yes	No	No	No	Brisk	No	No	Yes	No	Yes	No	NA	c.466G > A, p. E156K
11, G	F	44	49	5	4	2	Yes	No	Yes	No	Brisk	No	Yes	No	No	No	Mild	_	c.230G > A, p.C77Y
12, H	F	30	47	17	2	3	Yes*	No	Yes	No	Brisk	Yes	Yes	No	No	No	No	+	c.1381G > A, p.A461T
13, I	M	1	7	6	21	2	Yes	Yes	Yes	SP	Brisk	Yes	Yes	No	Yes	Yes	ID	_	c.1308C > G, p.Y436*
14, J	F	0	14	14	31	4	Yes	Yes	Yes	GP	Brisk	Yes	Yes	NE	Yes	No	ID	_	c.419G > A, p.R140Q

^*^Spasticity

*LL *lower limbs, *NA *not available, *NE *not evaluable, *SP *saccadic pursuit, *SS *Slow saccades, *HS *hypometric saccades, *ID *intellectual disability, *SARA *scale for the assessment and rating of ataxia, *SDFS *spinocerebellar degeneration functional score

Excluding the pediatric cases, mean SARA score was 11.5 ± 8.7. The mean progression rate was 0.94 ± 0.63 with disease duration of 14.7 years ± 11.8.

In the twelve adult-onset patients, the first symptom was gait ataxia in ten, while episodic ataxia and spasticity were the first manifestations in the remaining two. In the two patients with childhood onset, developmental delay and feeding difficulty were the presenting features.

Over the course of the disease, all patients developed gait ataxia, even though in Pt. 12 the phenotype was dominated by spasticity. Seven patients (50%) had abnormal ocular movements, consisting in saccadic pursuit in three, saccadic hypometria, slow saccades, nystagmus and gaze palsy in one each. Dysarthria and dysmetria were present in eleven (76%). Knee jerks were brisk in six (43%), associated with lower limb increased tone in four and Babinski signs in five. Vibration sense was decreased in three (21%). Dystonia and tremor were each present in three (21%). No patients presented bradykinesia and myoclonus. Sphincter disturbances were present in five (36%), erectile dysfunction in one. Three patients had mild cognitive impairment (21%), three intellectual disability (20%). Depression was present in two, one of whom was also psychotic (Table 1).

**Fig. 3:**
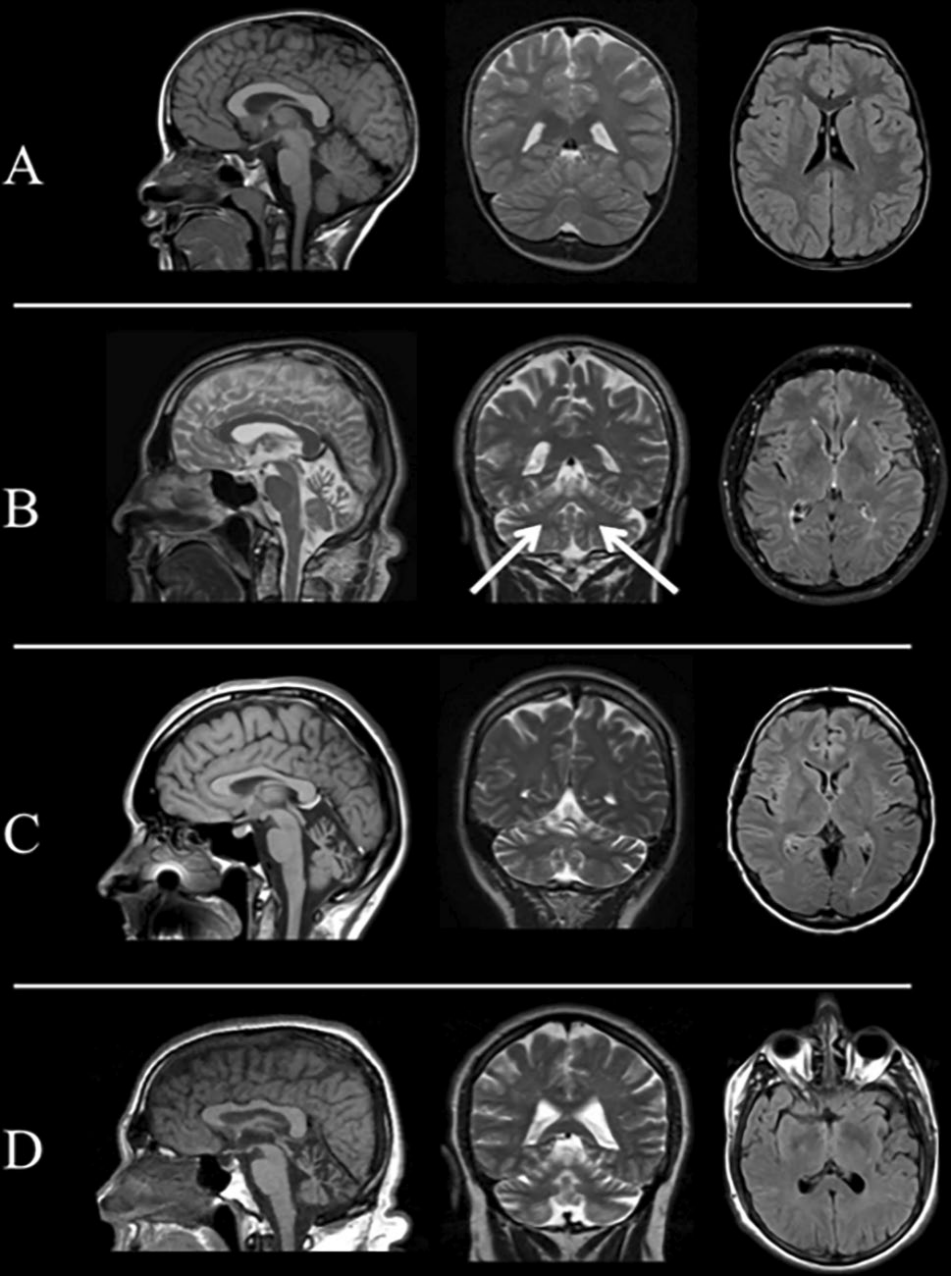
MRI: Representative brain MRI findings in SCA 14 patients. Brain MRI scans of four SCA14 patients (A: Pt. 13; B: Pt. 2; C: Pt. 8; D: Pt. 1). In the first two columns, from left to right, T1-weighted sagittal (with the exception, in B of a T2-weighted sequence) and coronal T2-weighted images showing the absence (**A**), the presence of moderate (**B**; **C**) and severe (**D**) cerebellar atrophy, respectively. In the third column axial Fluid Attenuated Inversion Recovery (FLAIR) images showing the absence of supratentorial signal changes, as well as preserved cerebral volumes (finding also visible in the other columns). The arrows in **B** indicate the presence of a mild T2-weighted hyperintensity affecting both dentate nuclei

Brain MRI scans were performed in 13/14 patients. In ten of the thirteen patients (77%), cerebellar atrophy was present, a percentage that increased to 91% (10/11 subjects) when considering adult-onset patients only. The degree of atrophy ranged from mild to moderate in almost all positive cases (9/10 subjects), with only one patient showing a severe degree of atrophy (Table1). The pattern of cerebellar atrophy was diffuse in more than half of the cases (6/10, 60%), while a pattern of more pronounced vermian and anterior lobe atrophy was found in the remaining patients. Infratentorial signal changes (mild dentate T2-hyperintensity) were found only in Pt. 2. No cases showed any significant supratentorial atrophy or signal abnormalities (Fig. 3).

Peripheral nerve conduction studies, performed in four patients (Pt 1, 6, 9, 11), were normal, as it was EMG, performed in two (Pt 6, 9).

We present in details the clinical presentation seen in four atypical cases.

Patient 8 is a 42-year-old Polish woman, without positive family history. Onset was at the age of 35 years with rare episodes of ataxia (two/year) lasting few days, sometimes associated with headache, without evident triggers. Since the age of 40, a mild ataxia became persistent and slightly progressive. Mild dysarthria was also present. SARA score was 5. Myoclonus, dystonia and tremor were absent. Cognition was normal (MMSE 28/30). Her MRI scan showed a moderate cerebellar atrophy, more marked in the anterior lobe. Other features included headache, both in association and independently from the ataxic episodes, and sinus tachycardia (Video 1).

Patient 12 is a 47-year-old woman, who presented gait difficulties since age 30. Family history was negative. At neurological examination, she had marked spasticity in the lower limbs, with increased lower limb tendon jerks and Babinski signs. Mild cerebellar symptoms (ataxia and dysmetria) were also present, without dysarthria. SARA score was 2. Cognition was normal. The brain MRI scan showed the presence of a mild, diffuse, cerebellar atrophy.

Patient 13 is a 7-year-old Filipino boy, born after an uneventful pregnancy and delivery. He had global developmental delay and walked unaided at 21 months. He never acquired language abilities and comprehension was limited. At the age of four, neurological examination revealed wide based gait and appendicular ataxia, speech limited to sounds, sialorrhea, prominent generalized jerky tremor, brisk knee jerks with increased lower limb tone and Babinski signs, dystonic posturing of the upper limbs (Video 2). At the last follow-up visit at age 7, there was slight improvement of the truncal ataxia and tremor, and worsening of dysmetria. SARA score was 21. The intellectual disability was severe. An extensive metabolic work-up including plasma amino acids, acylcarnitines, urinary organic acids, homocysteine, serum carbohydrate deficient transferrin analysis, plasma vitamin E, oxysterols, chitotriosidase activity, alpha-fetoprotein was negative. Plasma lactate was slightly increased. Karyotype, array-CGH and pathological expansion in *FMR1* were normal. Interestingly, his brain MRI scan (at the age of 7 years) was normal, without any abnormality both in terms of volume loss or signal changes.

Patient 14 is a 14-year-old girl, born at 35 weeks by cesarean delivery due to poor intrauterine growth and cardiotocographic alterations. Brain ultrasound showed a grade I intraventricular hemorrhage. Developmental delay and poor sucking were detected in the first months of life. On examination at the age of 5 years she presented with a spastic–ataxic gait needing support, gaze palsy and unintelligible speech. An extensive metabolic work-up, including filipin staining test, determination of serum beta-glucosidase, plasma amino acids, as well as urinary amino acids, organic acids, and pterins was normal. Karyotype, array CGH and mutations in the *UBE3A* gene were negative. Neurography was normal. She received the diagnosis of focal epilepsy with secondary generalization successfully treated with valproic acid. The clinical picture was slightly progressive. At the last follow-up at age 14 she had spastic ataxic gait needing support, gaze palsy, brisk knee jerks with increased lower limb tone and Babinski signs, limb dystonia and severe cognitive impairment SARA score was 29. Similar to Pt 13, her brain MRI scan was normal, with neither brain atrophy nor signal abnormalities. Longer neuroimaging follow-up will be required to ascertain involvement of brain structures. The trio analysis did not show other predictably or possibly pathogenic variants segregating in the families I (Pt 13) and J (Pt 14) and with clinical significance.

## Discussion

We describe fourteen patients in ten families harbouring variants of pathogenic significance in *PRKCG* gene*.* The frequency of SCA14 in our overall cohort of patients with inherited ataxia examined with TRP was 2.8% (and 8% in familial cases only), numbers that fall in the previously reported range (1–7%) [1–4]. Eight of the nine variants are missense, a finding in line with previous reports, where 84% of *PRKCG* disease-causing variants are missense. We highlight the presence in our series of the second nonsense variant reported thus far [7]. Amidst the nine identified variants, six are located in the regulatory domain, where lay more than 70% of SCA14-causing variants detected to date. We further identified three mutations in the catalytic domain, a protein region, where only seven pathogenic variants have so far been described (Fig. 1), in association with an apparently more complex phenotype [1, 6] a finding also seen in our patients.

The pathogenic mechanisms of PKCγ mutations are not completely understood. A gain of function hypothesis seems to be supported by in vitro and knock-in mouse studies, showing an increased PKCγ activity leading to aberrant dendritic development, neuronal death, mislocalization and aggregation effects [14–17]. Neurologically normal null-animals do not favour haploinsufficiency [18, 19]. A dominant-negative mechanism may be at least partially responsible for the disease caused by the non-sense mutation c.226C > T, p. R76* [7].

In our series, mean disease onset was at 32.6 years and the progression rate—when we do not take into account the pediatric cases—was 0.94 SARA point/year. After a mean disease duration of 14 years, only one patient was wheelchair bound, suggesting a non-particularly aggressive course of disease. Dysarthria and dysmetria were common; pyramidal signs (brisk knee jerks associated with increased lower limb tone and Babinski signs) were present in about one-third; focal dystonia in three. Mild cognitive impairment occurred in three, intellectual disability in three, marked in the two with childhood onset. Differently from the previously reported cases, in our series no patients presented myoclonus, and only one had nystagmus.

At MRI, we found the presence of cerebellar atrophy in 91% of the adult-onset patients, that ranged from mild to moderate, sometimes with a more prominent vermian and anterior involvement [2]. The normal MRI, in particular in children, does not exclude the development of cerebellar atrophy in the follow-up, since SCA14 natural history is not well known. Other infratentorial as well as supratentorial structures were spared. We only found a mild T2-weighted hyperintensity of both dentate nuclei in one patient (Pt. 2). This finding has already been described in other degenerative ataxias, such as ataxia with oculomotor apraxia (AOA) [20], SPG7 [21] and SCA48 [22]. Pieperhoff et al. (2020) [23] reported constant dentate hyperintensity in T2-weighted images in twenty SCA14 and nine AOA2 patients. However, this sign seems to be not specific and, at least in our series, not constant. Future multicentric studies are warranted to further expand the knowledge about the occurrence of this sign in SCA14.

Four patients have peculiar phenotypes, however. Patient 8 harbored the p.V138E mutation and started with episodic ataxia, followed by slightly progressive ataxia. This mutation has been already reported by Chelban et al. [2] in a patient in whom dystonia was the only associated feature. Other genetic and epigenetic factors may explain the different phenotypes. Thus, it is tentative to speculate that *PRKCG* might be added to the genes responsible of episodic ataxia. Patient 12 (c.1381G > A, p.A461T) had a predominant spastic phenotype. Even though increased knee jerks were present in one-third of the patients, a prominent pyramidal involvement has not been described so far. It is of interest that the responsible variant concerns the catalytic subunit, where mutations associated with more complex phenotypes have been described [2, 6]. In both cases, the mutations likely arose de novo, though this could not be formally proved, since parents were not available for DNA testing and clinical examination. Finally, two children (Pt. 13 and 14) showed an unusually severe phenotype with severe intellectual disability and speech limited to sounds, and non-acquisition of independent walking in Pt. 14. In both these pediatric cases, MRI proved to be normal, without volume loss or MRI signal changes. Patient 13 carried the second reported nonsense mutation (c.1308C > G, p.Y436*) and Pt. 14 harbored a novel missense (c.419G > A, p.R140Q), both falling in the regulatory domain. In both cases, the mothers carried the same mutations found in the children. Both have a normal neurological examination at the last follow-up, but we cannot exclude that they could show signs of the disease in the future. There are several reports of childhood onset of SCA14 [2–4, 6, 7, 24–28]. Unfortunately, the information about these cases is scanty and incomplete at most. One child was reported to have a very early onset with a delayed motor and speech development [24]. Information about the intellectual development is available in few and may vary from normal to mild impairment [2, 3, 6, 7]. Axial and multifocal myoclonus have been reported in few instances [25, 28], epilepsy [7] and neurological stability [25] in one case each. None seems so severe as Pt. 13 and Pt. 14. However, Pt. 14 had abnormal fetal growth. This event may be at least partially responsible for the severe neurological phenotype; on the other hand, the poor development of the fetus may also be a manifestation of *PRKCG* mutation.

In conclusion, we report seven novel, pathogenic and likely pathogenic variants in *PRKCG* gene, including the second nonsense reported so far. Four patients had peculiar phenotype (episodic ataxia, prevalent spasticity, severe infantile). Altogether, these findings may broaden the genetic and phenotypical aspects in SCA14.

## Supplementary Information

Below is the link to the electronic supplementary material.

Supplementary file1 (DOCX 16 KB) List of the 273 screened genes

Supplementary file2 (XLS 33 KB) This table lists predictions in silico and gnomAD frequencies for all the disease-causing variants identified in this study. We also included the multiple criteria for pathogenicity scoring in keeping with the guidelines of the American College of Medical Genetics and Genomics

Supplementary file3 (MP4 51642 KB) Pt. 8: a 42-year-old woman carrying the c.413T>A, p.V138E mutation and starting with episodic ataxia at the age 35. Since the age of 40, neurological examination shows very slight ataxia and instability in standing in tandem

Supplementary file4 (MP4 53443 KB) Pt. 13: a 4-year-old child carrying the c.1308C>G, p.Y436* nonsense mutation in the catalytic domain of PRKCG. Segment 1 shows gait instability and upper limb dystonia. Segment 2 and 3 show axial and appendicular jerky tremor, and upper limb dystonia, more evident on the right arm
