# Supplementary material for: Episodic ataxia and severe infantile phenotype in spinocerebellar ataxia type 14: expansion of the phenotype and novel mutations
Source: J Neurol. 2021 Jul 22;269(3):1476–84. doi: 10.1007/s00415-021-10712-5 (PMC8857164; doi:10.1007/s00415-021-10712-5)
Supplement: Supplementary file 1 — Supplementary file1 (DOCX 16 KB) List of the 273 screened genes [file 415_2021_10712_MOESM1_ESM.docx]

| **Supplementary Table 1.**  **List of the 273 screened ataxia genes.** | | | |  |  |  |
| --- | --- | --- | --- | --- | --- | --- |
| AARS2 | CACNB4 | EIF2B3 | KCNC1 | PDSS1 | SACS | TGM6 |
| ABCB7 | CAMTA1 | EIF2B4 | KCNC3 | PDSS2 | SAMD9L | THG1L |
| ABCD1 | CASK | EIF2B5 | KCND3 | PDYN | SCN1A | TINF2 |
| ABHD12 | CC2D2A | ELOVL4 | KCNJ10 | PEX10 | SCN2A | TMEM138 |
| ACO2 | CCDC88C | ELOVL5 | KCTD7 | PEX16 | SCN8A | TMEM216 |
| ADCK3 | CD40LG | ERCC4 | KIF1A | PEX2 | SCYL1 | TMEM231 |
| ADGRG1 | CDK5 | ERCC8 | KIF1C | PEX6 | SEPSECS | TMEM237 |
| AFG3L2 | CEP104 | EXOSC3 | KIF7 | PEX7 | SETX | TMEM240 |
| AHDC1 | CEP290 | EXOSC8 | LAMA1 | PHYH | SIL1 | TMEM67 |
| AHI1 | CEP41 | FA2H | LMNB2 | PIK3R5 | SLC17A5 | TOP1 |
| ALDH5A1 | CHMP1A | FARS2 | LYST | PLA2G6 | SLC1A3 | TPP1 |
| ALG3 | CLCN2 | FASTKD2 | MARS2 | PLEKHG4 | SLC25A46 | TRNT1 |
| ALG6 | CLN5 | FGF14 | MED17 | PLP1 | SLC2A1 | TRPC3 |
| AMACR | CLN6 | FLVCR1 | MFN2 | PMM2 | SLC33A1 | TSEN2 |
| AMPD2 | CLN8 | FMR1 | MFSD8 | PMPCA | SLC35A2 | TSEN34 |
| ANO10 | CLP1 | FOLR1 | MKS1 | PNKP | SLC52A2 | TSEN54 |
| APOB | COA7 | FXN | MMACHC | PNPLA6 | SLC6A19 | TTBK2 |
| APTX | COQ2 | GALC | MME | POLG | SLC9A1 | TTC19 |
| ARL13B | COQ4 | GAN | MRE11A | POLR3A | SLC9A6 | TTC21B |
| ARSA | COQ9 | GBA2 | MTPAP | POLR3B | SMPD1 | TTPA |
| ATCAY | COX20 | GBE1 | MTTP | PPT1 | SNAP25 | TUBB3 |
| ATG5 | CP | GDAP2 | MVK | PRICKLE1 | SNX14 | TUBB4A |
| ATL1 | CSPP1 | GFAP | NAGLU | PRKCG | SPAST | UBA5 |
| ATM | CSTB | GJB1 | NDUFS1 | PRNP | SPG11 | UBR4 |
| ATP13A2 | CTBP1 | GJC2 | NDUFS7 | PRPS1 | SPG7 | UCHL1 |
| ATP1A3 | CTSD | GLB1 | NEU1 | PRRT2 | SPTBN2 | VAMP1 |
| ATP2B3 | CWF19L1 | GOSR2 | NOL3 | PSAP | SRD5A3 | VARS2 |
| ATP7B | CYP27A1 | GRID2 | NOP56 | PSEN1 | STS | VLDLR |
| ATP8A2 | CYP7B1 | GRM1 | NPC1 | PTF1A | STUB1 | VRK1 |
| BEAN1 | DARS | HARS | NPC2 | PTRH2 | STXBP1 | VWA3B |
| BRAT1 | DARS2 | HARS2 | NPHP1 | QARS | SURF1 | WDR81 |
| BRF1 | DDHD2 | HEXA | OFD1 | RAB3GAP1 | SYNE1 | WFS1 |
| C10ORF2 | DKC1 | HEXB | OPA1 | RARS | SYNE2 | WWOX |
| C12ORF65 | DNAJC19 | HIBCH | OPA3 | RARS2 | SYT14 | XPA |
| C5ORF42 | DNAJC3 | HSD17B4 | OPHN1 | RELN | TBC1D23 | XRCC4 |
| C9ORF72 | DNMT1 | INPP5E | OTUD4 | RNF170 | TCTN1 | ZFYVE26 |
| CA8 | EEF2 | ITPR1 | PAX6 | RNF216 | TCTN2 | ZFYVE27 |
| CACNA1A | EIF2B1 | KCNA1 | PDE6D | RPGRIP1L | TCTN3 | ZNF423 |
| CACNA1G | EIF2B2 | KCNA2 | PDHA1 | RUBCN | TDP1 | ZNF592 |
